# Support for smoke-free policy among restaurant owners and managers in Ulaanbaatar, Mongolia

**DOI:** 10.1136/tc.2009.030486

**Published:** 2009-10-01

**Authors:** S-H Chang, V Delgermaa, K Mungun-Ulzii, N Erdenekhuu, E Odkhuu, S-L Huang

**Affiliations:** 1 Institute of Public Health, National Yang-Ming University, Taipei, Taiwan; 2 Department of Preventive Medicine of School of Public Health, Health Sciences University of Mongolia, Ulaanbaatar, Mongolia; 3 Cardiovascular Disease Department, Shastin’s Central Hospital, Ulaanbaatar, Mongolia; 4 Department of Pathophysiology, School of Biomedicine, Health Sciences University of Mongolia, Ulaanbaatar, Mongolia

## Abstract

**Objectives:**

Exposure to second-hand smoke (SHS) is widespread in restaurants in Ulaanbaatar, the capital city of Mongolia. While a smoke-free policy is the most effective way of protecting restaurant workers and customers from SHS, this has not been well accepted in Mongolia. Furthermore, little is known about restaurants’ attitude toward the smoke-free policy.

**Methods:**

A cross-sectional survey directed to restaurant owners or managers was conducted in 475 representative restaurants in Ulaanbaatar. Face-to-face interviews using a questionnaire and on-site observation were performed.

**Results:**

Only 29.3% of the restaurants claimed to prohibit smoking; none of the remaining had any protection toward SHS, and half of the restaurants estimated that more than 20% of customers would smoke inside. None of them had visible “no smoking” signs and the majority never received complaints about SHS. Despite the generally high level of knowledge of the health effects of SHS, of the 336 restaurants that were not smoke free, only 25.9% expressed that they planned to take action in the near future. By contrast, 87.8% of restaurants would support the government if it asked all restaurants to ban smoking. Multivariate analysis identified that restaurants having menus in foreign languages, selling cigarettes and predicting business decline were less likely to support the government smoke-free policy.

**Conclusions:**

This survey demonstrates that restaurants owners and managers were reluctant to take action on their own, but would support government policy. The government can assume a stronger role first by revising the law on tobacco control following the Framework Convention on Tobacco Control guideline.

Smoking in low and middle income countries is a major concern in global public health.^1^ In countries without comprehensive tobacco control policies, extensive tobacco product marketing and the influence of the tobacco industry may have important adverse public health consequences.^2^ ^3^ In response to this situation, the World Health Organization (WHO) adopted the Framework Convention on Tobacco Control (FCTC) in 2003, which prescribed “protection from exposure to tobacco smoke in indoor workplaces, public transport, indoor public places and, as appropriate, other public places”.^4^ In addition to reducing harms from second-hand smoke (SHS) exposure, a smoke-free policy also reduces the amount of tobacco use among smokers, encourages smokers to quit and prevents youth from taking up smoking.^5^ ^6^

Mongolia is located between Russia and China with a population of 2.6 million, and about 1 million people reside in Ulaanbaatar, the capital city.^7^ Mongolia is categorised by the United Nations Development Program as a “medium human development”, with average life expectancy of 66.3, adult literacy rate of 97.4% and gross domestic product (GDP) per capita of $2887.^8^ According to the 2005 Mongolian STEPwise approach to surveillance (STEPS) survey, daily and occasional tobacco use in men aged 15 and above were 43.1% and 5.3%.^9^ A previous survey in Ulaanbaatar, however, revealed a much higher adult smoking rate of 67% in men and 21% in women.^10^ The 2003 Global Youth Tobacco Survey showed that among adolescents 73.9% of boys and 71.7% of girls reported being exposed to second-hand smoke, with parental smoking prevalence 58%.^11^

Mongolia ratified the WHO FCTC in February 2004, and subsequently revised the Law on Tobacco Control in 2005. Article 9.2 of the Law stipulates that “smoking shall be restricted in other areas except specially designated areas of …public eateries, shops, entertainment and service places, bars and restaurants”, with relatively low fines (approximately US $3.50–$7.00) for citizens who violated that article. The Law further requires that “public eatery with two or more service hall [sic], shall designate a special service hall for non-smokers”. Also, “business entities and organisations indicated in 9.2 shall place signs and warnings at places where smoking is allowed or prohibited…” These regulations do not comply with the “Guidelines for the implementation of [FCTC] Article 8”, which recommend “total elimination of smoking and tobacco smoke in a particular space or environment in order to create a 100% smoke free environment”.^12^ The Ulaanbaatar city government started to host “best practice award” campaigns to promote health behaviour in 2002, and included a “smoke-free restaurants award” for the first time in 2009. However, the survey reported in this paper, undertaken in early 2008, shows that most restaurant owners and managers were not aware of the Law on Tobacco Control.

Restaurants are one of the workplaces where workers are most exposed to SHS.^13^ A smoke-free policy in restaurants would drastically improve air quality, reduce exposure to second-hand smoke and improve the health of workers.^14^ ^15^ ^16^ Furthermore, a smoke-free policy in restaurants and bars may help to advance the social unacceptability of tobacco use, and reduce cigarette consumption among the general public.^17^

Information regarding public opinion and support, as well as factors affecting people’s attitudes, may help the government in formulating policies. A survey prior to the legislation for smoke-free policies in all restaurants and bars in Hong Kong indicated that there had been high level of public support.^18^ Even starting with less encouraging levels of support, it has been demonstrated that public support for smoke-free policies increased after smoke-free policies were implemented in four countries with different smoking rates and varying degrees of policy stringency.^19^ Restaurant owners and managers are important stakeholders in smoke-free policies, as they must take into consideration economic factors, customer demands and considerations, in addition to health issues.^20^ A study in China found that 60.6% of restaurant owners thought that banning smoking would decrease profits, but 57.5% also felt that smoke-free restaurants were “feasible” or “very feasible”.^21^ Such initial ambivalence is not unusual, as positive attitudes toward smoke-free policies among restaurant and bar owners increases after implementation.^22^ ^23^

This study aimed to describe the current situation of smoking policy among restaurants in Ulaanbaatar, as well as knowledge and attitudes about SHS, and support for a smoke-free policy among restaurant owners and managers.

## Methods

### Sampling

This cross-sectional survey was aimed at the owners or managers of restaurants in Ulaanbaatar. The restaurants included various types of formal and casual dining facilities but did not include outdoor diners, bars, or clubs. The owners were the preferred persons to interview, and managers or senior staff were interviewed if owners were unavailable or unwilling after two approaches. In the absence of a complete list of all restaurants, systematic cluster sampling was used to obtain a representative sample. Based on a 1:10 000 map of Ulaanbaatar published in 2007, the city was divided by major avenues into 3 approximately concentric areas: the central area, surrounding business/residential area and the outer residential area. The 3 areas were further divided by major roads into 22, 36 and 48 blocks, respectively. One block was randomly chosen from each area, and interviewers then surveyed every street to locate and interview owners/managers at all eligible restaurants thus located. The process was repeated 8 times until the total number of successful interviews exceeded 400. Each block in the central, surrounding and outer area contained on average 24.7, 24.1 and 16.8 restaurants, respectively. There were 533 restaurants in the 24 blocks selected for sampling, and 490 (91.9%) agreed to participate. Except for 10 unfinished interviews and 5 owners who could not communicate fully in Mongolian, 475 (89.1%) successful interviews were completed.

### The interview

A structured questionnaire was used in face-to-face interviews, which included six parts: sociodemographic data about interviewees, their personal smoking behaviour, restaurant characteristics (type, size, cigarette or liquor sale, menu in local and/or foreign language), current situation and policies regarding customer smoking, knowledge and attitudes towards SHS, and difficulties encountered or anticipated with adopting a smoke-free policy. The definition of “smoke-free restaurants” was printed on the questionnaire and read to the interviewee when it first appeared in the questions: ““Smoke-free restaurant” means total ban of smoking at any place inside the restaurant including dining section, kitchen and restroom”. Likert items were used for knowledge and attitude questions: “strongly agree”, “agree”, “neutral”, “disagree” and “strongly disagree”; the first two responses were considered positive in analysis. There were two open-ended questions in the questionnaire that asked about reasons why they did or did not support the smoke-free policy, and the difficulties they expected in implementing the policy. Responses to the two questions were recorded in English by the interviewers. Keywords were identified from their responses and grouped into different themes.

The questionnaire originally in English was translated into Mongolian. The back-translation and results from 35 pretest questionnaires were reviewed by local experts from the Health Sciences University of Mongolia, Ministry of Health, World Health Organization office and Public Health Professionals Association of Mongolia. A written consent form was presented to the interviewees before each interview. This study was approved by the Ethics Committee of Health Sciences University of Mongolia. The survey was carried out in January to May 2008.

### Observation record

The interviewers observed for the following outside and inside of restaurants: tobacco advertisements, “no smoking” signs, smoking areas, smoking rooms, cigarette butts on the ground, ashtrays, any person smoking, and functional objects such as ashtrays or clocks provided by tobacco companies. The observations outside the restaurant included the outer wall, the shop sign, the outer side of the front door and the immediate vicinity of the front door.

### Statistical analysis

The complex samples procedure in the SPSS V. 17.0 software package (SPSS, Chicago, Illinois, USA) was used to account for the stratified and clustered nature of sampling. The sampling weight for clusters in each stratum (central, surrounding, or outer area of the city) was calculated based on the ratio of number sampled versus the total number of restaurants, which was estimated by the average number of restaurants in the blocks sampled and the total number of blocks. To analyse the association of personal and restaurant characteristics with supporting the smoke-free policy, odds ratios for each variable were estimated by the complex samples crosstabs. Multivariate regression was used to estimate the odds ratio while controlling for relevant variables by the complex samples logistic regression procedure.

## Results

### Current status of smoking policy among restaurants

The 475 restaurants included in the study were distributed in various neighbourhoods in Ulaanbaatar, ranging from downtown fine dining restaurants serving tourists and business people to local establishments serving primarily local residents in residential, industrial and ger (yurt) areas. The number of staff varied from 1 to 80. Selected characteristics of the interviewees and restaurants are shown in table 1. The interviewees were mostly women, restaurant owners, relatively young and holding college degrees. The mean (standard error (SE)) prevalence of current daily smokers among staff was 63.5% (5.0%) in men and 21.3% (2.3%) in women. Many (283, 59.7%) sold liquor in the restaurants, and 279 (58.7%) sold cigarettes as well, but only 33 (11.8%) agreed that cigarette sales were an important source of income for the restaurant.

**Table 1:** Distribution of personal and business characteristics of the 475 restaurant owners/managers interviewed; percentage (standard error (SE)) supporting governmental smoke-free policy are shown, with odds ratio estimations

	Support government smoke-free policy, n (% (SE))	Odds ratio
Sex:		
Male	104 (77.5 (4.5))	0.34**
Female	371 (90.9 (1.2))	
Age:		
⩾36	191 (90.8 (1.7))	1.59
⩽35	284 (86.1 (1.5))	
Education:		
College	308 (87.1 (1.5))	0.78
Below college	167 (89.7 (1.9))	
Role:		
Manager	185 (88.3 (2.2))	1.06
Owner	290 (87.8 (1.8))	
Smoking status:		
Smoker	145 (80.2 (2.9))	0.38**
Non-smoker	330 (91.4 (1.5))	
Restaurant location:		
Residential/industrial	304 (89.1 (1.3))	1.32
Tourist/business	171 (86.0 (3.0))	
Restaurant type:		
Fine dining	269 (82.6 (1.9))	0.26**
Family diner	206 (94.9 (1.5))	
Menu in foreign language:		
Yes	152 (79.8 (2.9))	0.35**
No	323 (91.8 (1.1))	
Selling liquor:		
Yes	284 (81.4 (1.7))	0.11**
No	191 (97.6 (0.9))	
Selling cigarettes:		
Yes	279 (81.8 (1.8))	0.15**
No	196 (96.9 (1.1))	
Customers smoke, %:		
>20%	225 (83.1 (1.8))	0.40**
⩽20%	250 (92.4 (1.4))	
Advertisement:†		
Yes	56 (84.1 (4.5))	0.69
No	419 (88.5 (1.5))	
Effect on business:		
Decline	178 (79.2 (2.8))	0.27**
No change or increase	297 (93.3 (1.4))	

*p<0.05; **p<0.01; †presence of indoor tobacco product advertisement or functional objects carrying tobacco company logos.

The current policies and practices regarding customer smoking are shown in table 2. The majority of workers and customers in restaurants were not protected from SHS. Based on interviewers’ observations, only 29 (6.1%) restaurants had any “no smoking” signs posted outside or in the restaurant, although 139 (29.3% (2.3%)) said that the restaurants did not permit smoking inside the restaurants. Of the remaining 336 restaurants that allowed smoking, only 43 had a clearly designated smoking area, but none of them had complete physical or ventilation separation from the non-smoking area. Customer smoking is common, with almost half of restaurants estimating that more than 20% of their customers would smoke in the restaurants. The majority (90.6% (1.3%)) had never received any complaints from the customers about SHS in the past 6 months, and only nine (1.9% (0.5%)) reported complaints about SHS once or more per week. The Law on Tobacco Control is the principal legal instrument for regulating tobacco use in Mongolia, but only a third (33.2% (2.3%)) of the owners/managers had heard about the Law, and only 16.2% said that they knew its regulations. Many of those who gave descriptions of the regulation of the Law said incorrectly that smoking was totally prohibited in restaurants.

**Table 2:** Status of current policies and practices regarding smoking in the restaurants, and expected effects on business by the smoke-free policy (n = 475)

	n (% (standard error))
Separation into smoking and non-smoking areas:	
Smoke-free restaurant	139 (29.3 (2.3))
No separation (not even signs)	293 (61.7 (1.3))
Incomplete separation	43 (9.0 (0.6))
Complete separation	0 (0.0)
Estimated percentage of customers who smoked during dining:	
Less than 5%	172 (36.2 (2.4))
5% to 20%	78 (16.4 (1.5))
More than 20%	225 (47.4 (2.2))
Complaints about second-hand smoke in the past 6 months:	
Never	430 (90.6 (1.3))
Less than once a week	36 (7.5 (1.2))
1–4 times per week	7 (1.4 (0.5))
Almost every day	2 (0.5 (0.3))
Heard of Law on Tobacco Control:	
Never	317 (66.8 (2.8))
Yes	158 (33.2 (2.8))
Knew regulations of the Law on Tobacco Control:	
Nothing	398 (83.8 (2.3))
Something	77 (16.2 (2.3))
How do you think restaurant business will be affected if it becomes smoke free?	
No change	252 (53.1 (2.3))
Decrease	178 (37.5 (2.4))
Increase	9 (1.9 (0.7))
Unclear	36 (7.6 (1.3))
If the business decreased, how much would the decline be? (n = 178)	
Less than 25%	50 (28.1 (3.9))
26% to 50%	70 (39.3 (4.3))
51% to 75%	22 (12.3 (3.0))
76% to 100%	14 (7.9 (2.4))
Unclear	22 (12.3 (3.0))

### Knowledge regarding SHS and attitudes toward smoke-free policy

Notwithstanding the lack of knowledge regarding legal regulations and the lax smoking policies, most restaurant owners/managers had accurate knowledge about health effects of SHS (table 3). For example, 98.3% agreed that SHS would harm the health of children who were exposed, 82.6% agreed that SHS increased the risk of heart disease and 72.5% agreed that SHS increased the risk of lung cancer. Most (90.3%) thought that workers would be healthier if they worked in smoke-free restaurants. Likewise, their attitude towards SHS showed their personal willingness to avoid SHS. A majority (85.7%) said that they personally disliked SHS and 86.0% said they had the right to ask people not to smoke in restaurants. However, this claim to a right has not been actively asserted, as 39.3% agreed that it is OK for smokers to smoke around non-smokers in public places. Furthermore, the claim to a clean indoor air environment is further compromised by misunderstanding about the effect of designated smoking area, as 89.0% of participants agreed that “establishing the designated smoking area can protect non-smokers from the exposure to SHS”.

**Table 3:** Knowledge and attitude of restaurant owners/managers regarding the health effects of second-hand smoke (SHS), attitudes toward SHS and attitude toward smoke-free policy; shown in percentages

	Strongly agree	Agree	Neutral	Disagree	Strongly disagree
SHS increases the risk of heart disease in non-smokers	53.3	29.3	16.0	0.8	0.6
SHS increases the risk of lung cancer in non-smokers	41.1	32.8	22.4	2.9	0.8
SHS harms the health of children who are exposed	82.0	16.3	0.4	0.6	0.7
Employees working in smoke-free restaurants will be healthier	56.8	33.5	6.8	2.1	0.8
Establishing a designated smoking area can protect non-smokers from the exposure of SHS	53.7	35.3	5.1	2.7	3.3
I do not mind SHS	2.3	5.9	6.1	23.9	61.8
Non-smokers have the right to ask smokers to stop smoking in a restaurant	59.2	25.8	5.1	7.8	2.1
It is OK for smokers to smoke around non-smokers in public places	8.5	30.8	6.9	30.2	23.5
When families with children dine out, they prefer smoke-free restaurants	75.4	20.0	2.1	1.9	0.6
Smokers would not come to my restaurant if it is 100% smoke free	28.9	23.4	13.0	16.0	18.7
I will make this restaurant 100% smoke free in the near future (for restaurants that are not already smoke free, n = 336)	14.3	11.6	31.8	13.1	29.2
Would you support the government if it asked all restaurants to ban smoking inside the restaurant? (n = 475)	60.9	26.9	3.6	5.3	3.2
Would you support the government if it asked all restaurants to ban smoking inside the restaurant? (for restaurants that are not already smoke free, n = 336)	51.6	32.0	5.0	7.1	4.2

Of the restaurants that were not already smoke free, only 25.9% agreed that they would make their restaurants 100% smoke free in the near future. This reluctance towards a smoke-free policy was less pronounced when the restaurant did not have to act alone; 87.8% said that they would support the government if it asked all restaurants to ban smoking inside restaurants. One of the concerns was the adverse effect of a smoke-free policy on business: 178 (37.5% (2.4%)) thought that business would decline if the restaurant became smoke free. More than half of them (106/178, 59.5%) estimated the decline would be more than 25% (table 2).

### Factors associated with support of government smoke-free policy

The factors associated with support of the government regulation were analysed. First, univariate analysis was used to examine the association between personal and restaurant characteristics and “support for government smoke-free policy” (table 1). Owners/managers who had the following characteristics were less likely to support the governmental smoke-free policy: being male, being current smokers, working in fine dining restaurants (vs casual or family diners), having a menu in foreign languages (an indicator of serving tourists), having liquor/cigarette sales, estimating higher than 20% of customers were smokers, and being concerned about loss of business. Being the owner or manager of the restaurant was not significantly associated with support. In multivariate analysis, only having a menu in foreign language, cigarette sale and concern over business loss were independently associated with not supporting the government smoke-free policy (table 4).

**Table 4:** Multivariate analysis of factors associated with supporting the governmental smoke-free policy

Characteristic	OR (95% CI)
Sex (male vs female)	0.59 (0.23 to 1.56)
Smoking status (smoker vs non-smoker)	0.55 (0.26 to 1.15)
Job title (manager vs owner)	1.06 (0.52 to 2.14)
Menu in foreign language (yes vs no)	0.52* (0.31 to 0.87)
Selling cigarettes (yes vs no)	0.26* (0.13 to 0.71)
Customers smoke, % (>21% vs ⩽20%)	1.14 (0.64 to 2.04)
Anticipated policy effect on business (decline vs others)	0.48* (0.24 to 0.99)

Odds ratios (OR) were estimated by logistic regression, adjusted by variables shown in the table.

*p<0.05.

Two open-ended questions were asked. The first asked about reasons for supporting or not supporting the implementation of smoke-free policy in restaurants. Most remarks (439 in total) were in support of the smoke-free policy; over half mentioned concerns about the health of non-smokers and workers, and one-third said that smoke-free environments were more comfortable. Only 37 remarks expressed disapproval, and the reasons included “respect the right of smokers”, “bad for business” and “the government does not usually enforce the law”. The second question asked about the anticipated difficulties of implementing a smoke-free policy. There were more remarks anticipating some difficulties than those expressing certainty of implementation (261 vs 210). The owners/managers were mostly concerned that smokers would visit less or otherwise protest the regulation. Other reasons included “it is difficult to explain to the customers”, “we sell cigarettes”, “the Law would not be enforced” and “Mongolia is not ready for this”. There was no discernable disparity between the responses of owners versus managers.

## Discussion

This survey found that almost 70% of restaurants had no effective measures to protect workers and customers from SHS. Although generally the owners/managers had some knowledge regarding the health effects of SHS and showed willingness to protect non-smokers, not much action was taken. “No smoking” signs were not common, and none of the restaurants had effective separation between smoking and non-smoking areas. This cross-sectional survey describes the current situation rather than offering explanations, but several phenomena were relevant to the lack of action taken to protect against SHS exposure: rare complaints by non-smokers, misunderstanding that a designated smoking area was adequately protective, concerns over loss of the income from smokers, and weak regulation and implementation of the current Law on Tobacco Control. Under such a circumstance where people seemed to have become accustomed to pervasive SHS, it is not surprising that restaurant owners were not enthusiastic in pursuing a stricter smoking policy. However, it is encouraging to find out that most of the owners and managers would support the government if a clear smoke-free regulation were to come into force.

### Validity and potential biases

This survey tried to collect a representative sample from all restaurants in Ulaanbaatar, and was able to obtain a high response rate with generally satisfactory quality of interviews. Perhaps the most difficult term to understand in the questionnaire was “smoke-free policy”. Almost a third of participants in the pretests had difficulty in understanding the meaning of the term when it was shown in the same way as in governmental publications. Therefore a standard description of the policy was added and read to the interviewees when it first appeared in the questionnaire. Otherwise the questions were self-explanatory and were not reported to cause any problem. Some limitations of the survey are noted, however. The primary concern of this survey was restaurant owners and managers, but the support of customers and workers is equally important. Workers usually are exposed to SHS for a longer duration during working days, and would likely be concerned more about their own health and less about the business compared to owners. Therefore we could expect an even higher level of support from workers. We did not measure indoor pollution or biomarkers of exposure, therefore we could not document the actual exposure level, which is desirable when studying the effects of new regulation.

This survey was conducted via face-to-face interviews, and responses may have been subject to social desirability bias. The current situation (signs and smoking area) could be verified by direct observation by the interviewers. For example, the extent of smoking inside the restaurant could be substantiated by the presence of someone smoking in 178 (37.5%) restaurants during the course of interview, which took about 20–25 min in off-peak hours. The knowledge and attitude questions, however, were more likely to reflect their awareness of the researchers’ interests. In this survey it seemed that people had some awareness about the health effects of SHS, but we did not assess how serious they regarded the risks, which might be an important factor in their decision making. In addition, the response to questions regarding the percentage of customers who smoked and the percentage of business decline was a one-time estimation by the participants and could reflect the interviewee’s prior concern over tobacco use among customers, therefore it could be endogenous to the attitude toward a smoke-free policy.

### Reasons for the high level of support

We observed a remarkable gap between the lack of willingness to implement the smoke-free policy in their own restaurants and the high level of support for governmental policy. There could be several explanations for this. The support may indicate that most restaurant owners/managers sensed that SHS was a problem that needed to be addressed by the government. Secondly, social desirability bias could not be ruled out, but the interviewers did explain that the research was carried out by universities rather than the government. However, the support may also be founded on less genuine reasons. For example, the participants might not anticipate legislation for the smoke-free policy to be passed in a short time, and they also expressed doubts about whether the policy will be enforced by the government. In addition, most of the participants were not aware of the penalties for violating the regulations of the current Law on Tobacco Control, and it is likely that they have not given serious consideration to the future legal consequences of violating the smoke-free policy before they showed the support. However, it is citizens, rather than the restaurants owners, who are (in theory) penalised for violating the current law. Finally, the gap between supporting the policy and lack of voluntary action may stem from their concern over possible business decline. Though studies in different countries have shown that business were unaffected or even improved following implementation of smoke-free policies, this concern needs to be properly addressed.^24^ ^25^ ^26^ The findings in this study are similar to the results of a survey performed in two US cities, which found that most restaurant and bar owners and managers preferred the local government to require the city to be smoke free rather than to choose to go smoke free on their own.^26^ The author of that work argued that education campaigns encouraging individual restaurants to go smoke free would be less effective than persuading the government to enact an ordinance.

WHO recommended that a smoke-free policy, rather than one that relied on a designated smoking area, should be included in the law.^12^ While not many owners or managers would implement the smoke-free policy on their own, the majority did say they would support the government if it were to implement the smoke-free policy. The awareness of the health effects of SHS seemed to be enough, and most participants cited health concerns as their reasons for supporting the smoke-free policy. However, many of them misunderstood the effectiveness of a designated smoking area and very few of them had heard about the smoke-free policy, or the national Law on Tobacco Control. The results of this survey indicate the need for government to communicate with the public about the benefits and ways of implementing the smoke-free policy, and the legislators need not assume that business owners would necessarily be against this policy.

**What this paper adds**

- A smoke-free policy is the most effective way to prevent the health effects of second-hand smoke (SHS). Without law enforcement, restaurants owners and managers are often reluctant to implement the smoke-free policy, because the concern for health is contradicted by the concern about decline in business. There has not been sufficient understanding of the knowledge level and attitudes of restaurant owners and managers regarding smoke-free policy, particularly among middle-income countries.
- This survey in Ulaanbaatar, Mongolia, showed that restaurant owners and managers generally have adequate knowledge about the adverse effects of SHS, but they over-rated the usefulness of designated smoking areas. Few restaurants would take action to make their own restaurant smoke free, particularly those having tourist customers, selling cigarettes and predicting business decline following a smoking ban. In spite of the apparent resistance, most restaurants would support a governmental smoke-free policy. The results suggest that the Law on Tobacco Control should be revised, and that government should assume the leading role in tobacco control.

### Policy implications

During the interview some participants expressed the concern that the government has not enforced the law on tobacco control in the past. This indicated the need for more political will on the governmental side. Based on interviews with US state and local government officials, it was found that the clean indoor air laws were not systematically enforced by state or local authorities, but were largely self-enforcing. People voluntarily complied with the law largely because of changing social norms regarding appropriate smoking behaviour.^27^ However, the situation may be different in Mongolia. According to the community readiness model, the community has to progress through awareness and several preparation stages in order to create an environment in which legislation can take place, and the initial stages are largely awareness-creating in nature.^28^ ^29^ We have identified some characteristics of restaurants that are less likely to support a smoke-free policy, notably those that are selling cigarettes, those that are tourist-oriented and those that expect a decline in business. Educational programs and campaigns could be directed to demonstrate the low cost and effectiveness of implementing a smoke-free policy, but most importantly, the minimal effect on business should be stressed.

In conclusion, the survey found that exposure to SHS is widespread in restaurants in Ulaanbaatar. Although restaurants owners and managers recognised the health hazards of SHS, not many of them understood that a smoke-free policy was the most effective way to provide protection to customers and workers. An important finding was that although restaurants hesitated to take actions, likely due to concerns over business, most of them would support a government smoke-free policy. We suggest that the Law on Tobacco Control should be revised, following the FCTC guidelines, and that government assume the leading role in tobacco control. This survey is the first to investigate the extent of SHS exposure and smoking policies among restaurants in Mongolia. The results may provide useful information for the government, and could serve as a reference for future follow-up.

## References

[1] Jha P Chaloupka FJ The economics of global tobacco control. BMJ 2000;321:358–61 10926598 10.1136/bmj.321.7257.358 PMC1118333

[2] Mackay JM The tobacco industry in Asia: revelations in the corporate documents. Tob Control 2004;13:ii1–3 15564211 10.1136/tc.2004.010082 PMC1766157

[3] Sebrie E Glantz SA The tobacco industry in developing countries. BMJ 2006;332:313–14 16470028 10.1136/bmj.332.7537.313 PMC1363897

[4] World Health Organization WHO Framework Convention on Tobacco Control. Geneva, Switzerland: WHO, 2003 10.1136/bmj.327.7407.154 PMC1126513 12869461

[5] Siegel M Albers AB Cheng DM Effect of local restaurant smoking regulations on progression to established smoking among youths. Tob Control 2005;14:300–6 16183980 10.1136/tc.2005.012302 PMC1748108

[6] Hopkins DP Briss PA Ricard CJ Reviews of evidence regarding interventions to reduce tobacco use and exposure to environmental tobacco smoke. Am J Prev Med 2001;20:16–66 11173215 10.1016/s0749-3797(00)00297-x

[7] Mongolian Ministry of Health Health Indicators, 2006. Ulaanbaatar, Mongolia: Ministry of Health, 2007

[8] United Nations Development Program Human Development Indices. New York, USA: United Nations Development Program, 2008

[9] World Health Organization Mongolian STEPS Survey on the prevalence of noncommunicable disease risk factors 2006. Geneva, Switzerland: WHO, 2006

[10] Adventist Development and Relief Agency Evaluation Report of Tobacco Project (2000–2004). Ulaanbaatar, Mongolia: Adventist Development and Relief Agency, 2004: 1–35

[11] Rudatsikira E Siziya S Dondog J Prevalence and correlates of environmental tobacco smoke exposure among adolescents in Mangolia. Indian J Pediatr 2007;74:1089–93 18174643 10.1007/s12098-007-0203-y

[12] World Health Organization Guidelines for the implementation of Article 8, protection from exposure to tobacco smoke. Geneva, Switzerland: WHO, 2007

[13] Wortley P Caraballo RS Pederson LL Exposure to secondhand smoke in the workplace: serum cotinine by occupation. J Occup Environ Med 2002;44:503–9 12085475 10.1097/00043764-200206000-00010

[14] Goodman P Agnew M McCaffrey M Effects of the Irish smoking ban on respiratory health of bar workers and air quality in Dublin pubs. Am J Respir Crit Care Med 2007;175:840–5 17204724 10.1164/rccm.200608-1085OC

[15] Semple S Creely KS Naji A Secondhand smoke levels in Scottish pubs: the effect of smoke-free legislation. Tob Control 2007;16:127–32 17400951 10.1136/tc.2006.018119 PMC2598470

[16] Allwright S Paul G Greiner B Legislation for smoke-free workplaces and health of bar workers in Ireland: before and after study. BMJ 2005;331:1117–21 16230313 10.1136/bmj.38636.499225.55 PMC1283274

[17] Alamar B Glantz SA Effect of increased social unacceptability of cigarette smoking on reduction in cigarette consumption. Am J Public Health 2006;96:1359–63 16809588 10.2105/AJPH.2005.069617 PMC1522108

[18] Lam TH Janghorbani M Hedley AJ Public opinion on smoke-free policies in restaurants and predicted effect on patronage in Hong Kong. Tob Control 2002;11:195–200 12198268 10.1136/tc.11.3.195 PMC1759014

[19] Fong GT Hyland A Borland R Reductions in tobacco smoke pollution and increases in support for smoke-free public places following the implementation of comprehensive smoke-free workplace legislation in the Republic of Ireland: findings from the ITC Ireland/UK Survey. Tob Control 2006;15:iii51–8 16754947 10.1136/tc.2005.013649 PMC2593063

[20] Johnson HH Becker C Inman L Why be smoke-free? A qualitative study of smoke-free restaurant owner and manager opinions. Health Promot Pract 2008. In press. 10.1177/1524839907309866 18372432

[21] Zheng P Fu H Li G Smoke-free restaurants in Shanghai: should it be mandatory and is it acceptable? Health Policy 2009;89:216–24 18621430 10.1016/j.healthpol.2008.06.002

[22] Tang H Cowling DW Stevens CM Changes of knowledge, attitudes, beliefs, and preference of bar owner and staff in response to a smoke-free bar law. Tob Control 2004;13:87–9 14985604 10.1136/tc.2003.004390 PMC1747827

[23] Thomson G Wilson N One year of smokefree bars and restaurants in New Zealand: impacts and responses. BMC Public Health 2006;6:64–72 16533408 10.1186/1471-2458-6-64 PMC1475576

[24] Cremieux P-Y Ouellette P Actual and perceived impacts of tobacco regulation on restaurants and firms. Tob Control 2001;10:33–7 11226358 10.1136/tc.10.1.33 PMC1763993

[25] Hammar H Restaurant owner perceptions of the effects of a smoking ban. Health Policy 2004;70:243–54 15364153 10.1016/j.healthpol.2004.04.003

[26] Hays S Secondhand tobacco smoke and municipal smokefree ordinances: attitudes of restaurant and bar owners and managers. J Drug Edu 2006;36:279–95 10.2190/Q416-72W4-7518-2505 17533802

[27] Jacobson PD Wasserman J The implementation and enforcement of tobacco control laws: policy implications for activists and the industry. J Health Politics Policy Law 1999;24:567–98 10.1215/03616878-24-3-567 10386327

[28] York NL Hahn EJ The community readiness model: evaluating local smoke-free policy development. Policy Politics Nursing Pract 2007;8:184–200 10.1177/1527154407308409 18178926

[29] Edwards RW Jumper-Thurman P Plested BA Community readiness: research to practice. J Commun Psychol 2000;28:291–307

